# The shadow of violence: How intimate partner violence shapes contraceptive and maternal health service use across 25 African countries

**DOI:** 10.1371/journal.pgph.0005470

**Published:** 2025-11-20

**Authors:** Alex Bawuah, Linus Baatiema, Michael Sarfo, Francis Appiah, Sanni Yaya

**Affiliations:** 1 School of Global Studies, Faculty of Social Science, University of Sussex, Brighton, United Kingdom; 2 Ghana Health Service, Upper West Regional Health Directorate, Wa, Ghana; 3 Centre for Migration, Security and International Relations, Simon Diedong Dombo, University of Business and Integrated Development Studies, Wa, Ghana; 4 L and E Research Consult Ltd, Wa, Upper West Region, Ghana; 5 School of Human and Health Sciences, University of Huddersfield, Huddersfield, United Kingdom; 6 Department of Health and Social Care, Coventry University, Coventry, United Kingdom; 7 School of Graduate Studies, Lingnan University, Tuen Mun, Hong Kong SAR; 8 Department of Social Sciences, Berekum College of Education, Berekum, Bono Region, Ghana; 9 Department of Population, Family and Reproductive Health, School of Public Health, Kwame Nkrumah University of Science and Technology, Kumasi, Ghana; 10 George Institute for Global Health, Imperial College London, London, United Kingdom; University of California, Los Angeles and University of Cape Town, South Africa, SOUTH AFRICA

## Abstract

Despite increasing efforts to improve reproductive health outcomes in sub-Saharan Africa (SSA), disparities persist in the use of modern contraceptives and maternal health services (MHS). Evidence suggests that exposure to intimate partner violence (IPV) may influence women’s health-seeking behaviours, yet few studies have examined this relationship across multiple SSA countries. Using cross-sectional data from the most recent Demographic and Health Surveys (2015–2024) in 25 SSA countries, we analyzed 122,144 women aged 15–49 with complete information on IPV, contraceptive use, and MHS utilization. The primary outcome variables were current use of any modern contraceptive method and adequate MHS use. IPV exposure was measured using standardized DHS indicators for emotional, physical, and sexual violence. Multivariable logistic regression models adjusted for socio-demographic covariates and survey design were used to examine associations. Overall, 29.2% of women used modern contraceptives, and 39.2% received adequate MHS. About 34.3% of women had experienced at least one form of IPV. Women who experienced emotional (AOR = 1.15; 95% CI: 1.11–1.20), physical (AOR = 1.14; 95% CI: 1.07–1.20), or sexual violence (AOR = 1.09; 95% CI: 1.03–1.15) were significantly more likely to use modern contraceptives than those who had not. However, they were less likely to receive adequate MHS: emotional (AOR = 0.95; 95% CI: 0.91–0.99), sexual (AOR = 0.88; 95% CI: 0.83–0.95), and at least one form of IPV (AOR = 0.94; 95% CI: 0.90–0.98). Key predictors such as parity, education, household wealth, residence, distance to a health facility, employment, and media exposure significantly influenced the outcomes. While exposure to IPV may increase contraceptive use, possibly as a protective strategy, it simultaneously reduces uptake of comprehensive maternal healthcare. Integrating IPV screening, counselling, and support services into reproductive and maternal health programs is critical for improving women’s health outcomes.

## Introduction

Intimate Partner Violence (IPV) is a pervasive human rights and public health issue with far-reaching consequences for women’s health and well-being, particularly across Africa. The World Health Organization estimates that nearly one in three women globally has experienced physical and/or sexual violence by an intimate partner, with Sub-Saharan Africa reporting some of the highest prevalence rates. IPV encompasses not only physical and sexual abuse but also psychological coercion, emotional abuse, and controlling behaviors, each of which can undermine women’s autonomy and compromise their reproductive health. A growing body of evidence links IPV to adverse maternal and reproductive outcomes, such as unintended pregnancies, miscarriage, sexually transmitted infections, and complications during childbirth [1–3].

In Africa, the threat of IPV intersects with deep-rooted structural inequalities, patriarchal gender norms, and overstretched health systems. Despite progress in expanding access to antenatal care (ANC), skilled delivery, and contraceptive services, maternal health indicators remain poor in many contexts. Sub-Saharan Africa still accounts for nearly two-thirds of all global maternal deaths [1]. Modern contraceptive methods, as defined by the DHS, include short-acting methods such as oral pills, injectables, and male/female condoms; long-acting reversible methods such as intrauterine devices (IUDs) and implants; and permanent methods including male and female sterilization. The diversity of these methods shows not only women’s ability to exercise reproductive autonomy but also the structural and health system barriers that may limit their access to preferred options. IPV acts as a significant barrier to maternal healthcare by restricting women’s mobility, decision-making capacity, and access to financial and informational resources [4]. Studies show that women exposed to IPV are less likely to initiate ANC early, deliver in health facilities, or use postpartum and family planning services [5], underscoring the urgent need for gender-sensitive interventions across the health sector.

The influence of IPV on maternal and reproductive health service use has been documented in various country-level studies. Aboagye et al. [6] observed that women exposed to partner violence were significantly less likely to attend ANC or deliver in a facility. Similar patterns were reported in Ethiopia [7], Kenya [8,9], and Nigeria [9], where IPV served as a barrier to timely and adequate healthcare utilization. Silverman et al. [10]found that IPV was linked with unmet need for contraception across multiple countries. In northern India and parts of Africa, Muluneh et al. [11] identified fear of violent repercussions from partners as a key factor deterring women from using contraceptives. Studies by Capaldi et al. [12] and Garcia-Moreno et al. [13] further emphasize the long-term consequences of IPV, including reproductive coercion, psychological trauma, and poor maternal outcomes.

Moreover, Abramsky et al. [14], using data from the WHO Multi-country Study, demonstrated that IPV is a consistent predictor of reduced health-seeking behavior among women, particularly in resource-constrained settings. Stockl et al. [15]and Makayoto et al. [16]confirmed these findings, underscoring that IPV acts as both a health issue and a social determinant that shapes women’s ability to access and utilize services. Despite this growing body of evidence, most of these studies are geographically limited, focusing on one or a few countries, and often emphasize physical and sexual violence while neglecting the broader spectrum of IPV especially psychological and controlling behaviors that are equally damaging but less visible.

Although Demographic and Health Survey (DHS) data are available for over 25 African countries, few studies have fully utilized this resource to examine inter-country differences, policy contexts, or the cumulative effects of multiple forms of intimate partner violence (IPV) on contraceptive and maternal health service (MHS) use. Most research focuses on single country analyses or emphasizes physical and sexual violence, with limited attention to emotional and controlling forms of IPV [5,17,18]. For example, studies by Teshale et al. [19], Aboagye et al. [2], and Muluneh et al. [11] analyze the link between IPV and antenatal care or facility delivery, but often within limited national contexts. Similarly, while Nakie et al. [4]and Tekeba et al. [5] highlight IPV prevalence across sub-Saharan Africa, they do not explore how such violence affects maternal and reproductive service uptake. This points to the need for broader, cross-country analyses that integrate various IPV forms and contextual variables to inform more responsive policy and programming.

This study, therefore, seeks to fill this critical gap by conducting a large-scale, cross-country analysis of the relationship between IPV and maternal healthcare and contraceptive service use in 25 African countries. Specifically, it investigates how exposure to various forms of IPV, physical, sexual, emotional, and controlling behaviors shapes women’s utilization of ANC, facility-based delivery, postnatal care, and family planning. By leveraging nationally representative data, the study also seeks to identify individual and contextual predictors and uncover regional patterns. Ultimately, the analysis demonstrates that the aim was achieved: the study provides robust, multi-country evidence on how IPV constrains reproductive and maternal healthcare, while offering insights to guide the development of gender-responsive health systems across Africa.

## Materials and methods

### Data source

The study used the most recent data from the Demographic and Health Surveys (DHS) of 25 countries in SSA conducted from 2015 to 2024. The countries included are Angola, Benin, Burkina Faso, Burundi, Cameroon, Côte d’Ivoire, Ethiopia, Gabon, Gambia, Ghana, Lesotho, Liberia, Madagascar, Malawi, Mali, Mauritania, Mozambique, Nigeria, Rwanda, Sierra Leone, South Africa, Tanzania, Uganda, Zambia, and Zimbabwe.

The DHS is a nationwide survey that is conducted in over 90 low- and middle-income countries worldwide and follows a consistent protocol and terminology across all countries [20]. It employs a structured questionnaire to gather information on various indicators of health, including maternal and child health, fertility, family planning utilisation, morbidity, and mortality [21]. The DHS uses a two-stage sampling technique to collect data, starting with the selection of enumeration areas based on each country’s sampling frame, followed by the selection of households from each enumeration area. Detailed information on the sampling and data collection methods can be found in the work of Aliaga and Ren [22].

The study employed the women’s dataset (IR file) from the DHS. The total sample from the pooled sample was 383,660 women aged 15–49. For this study, the sample is limited to women who responded to questions related to the outcome variables (modern contraceptive use and MHS utilisation) and independent variables of interest (IPV). Thus, after dropping all missing observations (via likewise deletion), we ended up with a sample size of 122,144 women.

### Variables

#### Outcome variables.

The study used two outcome variables: modern contraceptive use and MHS utilisation.

Modern contraceptive use was assessed using the DHS variable “v313”, which records the method of contraception currently employed by the respondent. The responses include: (i) no method, (ii) folkloric method, (iii) traditional method, and (iv) modern method. For this study, this variable was recoded into a binary outcome: 1 = using any modern method, and 0 = not using a modern method (i.e., no method, folkloric method, and traditional method).

MHS utilisation was assessed by creating a composite binary variable called adequate MHS, which captures whether a woman received essential maternal healthcare during her most recent pregnancy. The variable was coded 1 if the respondent: had at least four antenatal care visits, gave birth in a healthcare facility, and received postnatal care after delivery. Women who did not meet all three conditions were coded 0.

#### Independent variables.

The primary independent variable is intimate partner violence (IPV) exposure. This was assessed using four indicators of IPV: (i) emotional violence, (ii) physical violence, (iii) sexual violence, and (iv) at least one IPV.

Emotional violence was assessed using the DHS variable “d104”, which is coded “1” if the respondent reported experiencing any of the following acts committed by a current or former intimate partner: (i) said or did something to humiliate her in front of others, (ii) threatened to hurt or harm her or someone she cared about, or (iii) insulted her or made her feel bad about herself. The variable is coded “0” if none of these acts were reported.

Physical violence was assessed using the DHS variable “d107”, which is coded “1” if the respondents reported any of the following severe acts by an intimate partner: (i) kicked, dragged, or beat her up, (ii) tried to choke or burn her on purpose, or (iii) attacked her with a knife, gun, or other weapon. The variable is coded “0” if none of these acts were experienced.

Sexual violence was assessed using the DHS variable “d108”, which is coded “1” if the woman reported any of the following act by an intimate partner: (i) physically forced her into unwanted sex, (ii) physically forced her into other unwanted sexual acts, or (iii) forced her to perform sexual acts she did not want to. A response of “no” to all of these questions results in a code of “0”.

The last variable, “at least one IPV” is created from the three variables: emotional violence, physical violence, and sexual violence. This variable was coded “1” if the respondent reported experiencing at least one of the three forms of IPV. If the respondent reported no experience of all three forms, the variable was coded 0. This binary indicator captures whether the woman experienced any form of IPV, providing a broader measure of exposure”.

The following variables are included as covariates: age (15–19, 20–24, 25–29, 30–34, 35–39, 40–44, 45–49), education (no education, primary, secondary, higher), wealth (poorest, poorer, middle, richer, richest), residence (urban, rural), employment status (employed, unemployed), health insurance coverage (insured, uninsured), parity (0, 1–2, 3–5, ≥ 6), the distance to a healthcare facility is a problem (yes, no), frequency of reading newspapers/magazines (not at all, less than once a week, at least once a week, almost every day) frequency of watching television (not at all, less than once a week, at least once a week, almost every day) and frequency of listening to radio (not at all, less than once a week, at least once a week, almost every day). These variables were selected based on their significant association with the outcome variables in the literature [23–26] as well as their availability in the DHS dataset.

### Data analysis

The data were analysed with STATA version 18. Descriptive statistics were used to describe the sample as well as the prevalence of modern contraceptive use and adequate MHS across the 25 SSA countries. Multicollinearity was assessed by examining the variance inflation factor (VIF) and tolerance indices (TI) of the independent variables. Given the binary nature of the outcome variables, a binary multivariable logistic regression model [27,28] was employed to assess the association between IPV and the use of key reproductive healthcare services – modern contraceptive use and adequate MHS (analysis on the association between IPV and adequate MHS was limited to women with children). The survey design (sample weights, strata and primary sampling units) were taken into account in the regression analyses using the “svy” command in accordance with the DHS guide [29].

### Ethical consideration

This study used secondary data obtained from the Demographic and Health Surveys (DHS) Program. Access to the data was granted upon request through the DHS Program website (https://dhsprogram.com) after a formal application process. The dataset is anonymized and publicly available to researchers upon approval. As the data are de-identified and collected with informed consent by DHS, no additional ethical approval was required for this analysis. Further details on DHS ethical standards are available at: http://goo.gl/ny8T6X.

## Results

### Prevalence of modern contraceptive use and adequate MHS use

Table 1 reports the prevalence of modern contraceptive use and adequate MHS use in 25 SSA countries. The results show that Angola has the lowest prevalence of modern contraceptive use (8.51%, 95% CI: 7.91 - 9.16), whereas Zimbabwe has the highest prevalence of modern contraceptive use (63.28%, 95% CI: 62.03 - 64.51). Furthermore, Ethiopia has the lowest prevalence of adequate MHS use (11.43%, 95% CI: 10.35 - 12.61), whereas Ghana has the highest prevalence of adequate MHS use (74.46%, 95% CI: 72.61 - 76.23).

**Table 1:** Prevalence of modern contraceptive use and adequate MHS use.

	Modern contraceptives	Adequate MHS
**Country (year of survey)**	**% (95% CI)**	**% (95% CI)**
Angola (2015–2016)	8.51 (7.91 - 9.16)	19.88 (18.89 - 20.91)
Burkina Faso (2021)	33.83 (32.89 - 34.78)	63.04 (61.60 - 64.45)
Benin (2017–2018)	13.59 (12.62 - 14.63)	45.76 (44.07 - 47.46)
Burundi (2016–2017)	20.54 (19.63 - 21.48)	26.12 (25.00 - 27.27)
Côte d’Ivoire (2021)	19.94 (18.80 - 21.13)	39.15 (37.10 - 41.24)
Cameroon (2018)	16.78 (15.74 - 17.88)	44.30 (42.56 - 46.05)
Ethiopia (2016)	28.09 (26.83 - 29.39)	11.43 (10.35 - 12.61)
Gabon (2019–2021)	14.89 (13.62 - 16.25)	60.05 (57.66 - 62.39)
Ghana (2022)	27.19 (26.00 - 28.43)	74.46 (72.61 - 76.23)
Gambia (2019–2020)	16.49 (14.91 - 18.20)	62.57 (59.89 - 65.17)
Liberia (2019–2020)	27.93 (26.14 - 29.79)	66.33 (63.90 - 68.68)
Lesotho (2023–2024)	59.39 (57.38 - 61.37)	72.89 (69.21 - 76.29)
Madagascar (2021)	39.42 (38.19 - 40.67)	27.85 (26.40 - 29.34)
Mali (2018)	15.08 (13.91 - 16.33)	28.54 (26.79 - 30.35)
Mauritania (2019–2021)	11.91 (10.85 - 13.06)	22.56 (20.88 - 24.33)
Malawi (2015–2016)	54.72 (53.39 - 56.04)	23.77 (22.44 - 25.15)
Mozambique (2022–2023)	30.49 (29.15 - 31.86)	34.23 (32.20 - 36.32)
Nigeria (2018)	13.58 (12.88 - 14.31)	34.75 (33.61 - 35.91)
Rwanda (2019–2020)	55.06 (52.84 - 57.26)	33.21 (30.73 - 35.79)
Sierra Leone (2019)	20.48 (19.26 - 21.75)	72.69 (70.95 - 74.36)
Tanzania (2022)	30.68 (29.34 - 32.06)	37.20 (35.23 - 39.22)
Uganda (2016)	32.26 (31.21 - 33.32)	33.69 (32.43 - 34.96)
South Africa (2016)	53.56 (51.34 - 55.78)	72.00 (68.79 - 75.00)
Zambia (2018)	44.35 (43.21 - 45.48)	44.59 (43.24 - 45.94)
Zimbabwe (2015)	63.28 (62.03 - 64.51)	47.93 (46.32 - 49.54)

### Descriptive summary of the sample

**Fig 1:**
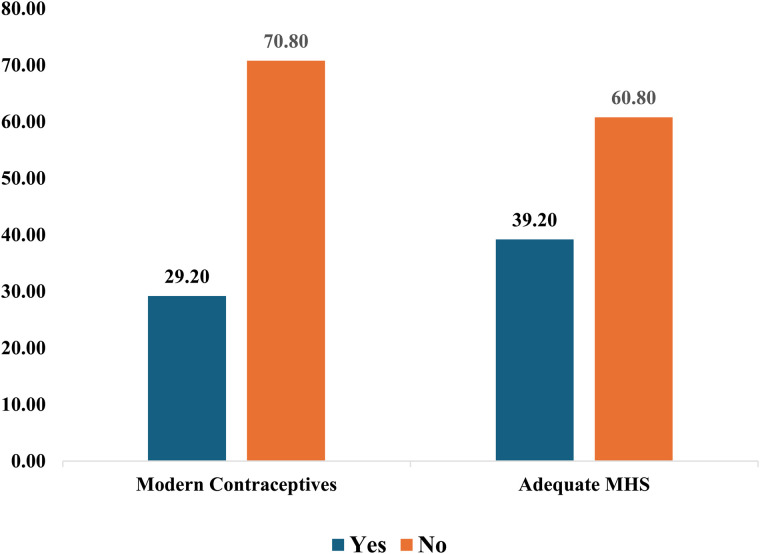
Levels of modern contraceptives and adequate MHS use in 25 SSA countries.

Fig 1 reports levels of modern contraceptive and adequate MHS use. It indicates 29.20% of the study sample uses modern contraceptives. Furthermore, 39.20% had adequate MHS.

Table 2 presents the results of the descriptive summary of the sample. It shows 29.32% of the women have experienced emotional violence by their husbands/partners, 10.87% have experienced sexual violence, 10.72% have experienced physical violence, and 34.29% have experienced at least one IPV.

**Table 2:** Descriptive summary of the sample.

Variables	Total sample N (%)	Modern contraceptives N (%)	Adequate MHS N (%)
**Emotional violence**			
No	86,336 (70.68)	24,447 (68.53)	21,275 (70.51)
Yes	35,808 (29.32)	11,225 (31.47)	8,899 (29.49)
**Sexual violence**			
No	108,867 (89.13)	31,317 (87.79)	27,331 (90.58)
Yes	13,277 (10.87)	4,355 (12.21)	2,843 (9.42)
**Physical violence**			
No	109,051 (89.28)	31,477 (88.24)	26,978 (89.41)
Yes	13,093 (10.72)	4,195 (11.76)	3,196 (10.59)
**At least one IPV**			
No	80,263 (65.71)	22,548 (63.21)	19,855 (65.80)
Yes	41,881 (34.29)	13,124 (36.79)	10,319 (34.20)
**Parity**			
None	9,514 (7.79)	1,314 (3.68)	
1 – 2 Children	40,613 (33.25)	13,052 (36.59)	12,777 (42.34)
3 – 5 Children	48,273 (39.52)	15,597 (43.72)	12,958 (42.94)
≥ 6 Children	23,744 (19.44)	5,709 (16.00)	4,439 (14.71)
**Age**			
15 - 19	8,208 (6.72)	1,748 (4.90)	1,596 (5.29)
20 - 24	21,600 (17.68)	6,697 (18.77)	6,349 (21.04)
25 - 29	25,434 (20.82)	8,175 (22.92)	8,039 (26.64)
30 - 34	23,420 (19.17)	7,740 (21.70)	7,033 (23.31)
35 - 39	19,032 (15.58)	5,840 (16.37)	4,680 (15.51)
40 - 44	13,868 (11.35)	3,695 (10.36)	1,935 (6.41)
45 - 49	10,582 (8.66)	1,777 (4.98)	542 (1.80)
**Educational level**			
None	41,396 (33.89)	7,293 (20.44)	7,963 (26.39)
Primary	41,110 (33.66)	13,388 (37.53)	9,058 (30.02)
Secondary	34,290 (28.07)	13,002 (36.45)	11,072 (36.69)
Higher	5,348 (4.38)	1,989 (5.58)	2,081 (6.90)
**Wealth**			
Poorest	27,237 (22.30)	5,891 (16.51)	5,248 (17.39)
Poorer	24,889 (20.38)	6,457 (18.10)	5,487 (18.18)
Middle	24,587 (20.13)	7,135 (20.00)	5,988 (19.84)
Richer	23,374 (19.14)	7,951 (22.29)	6,500 (21.54)
Richest	22,057 (18.06)	8,238 (23.09)	6,951 (23.04)
**Residence**			
Urban	44,104 (36.11)	13,849 (38.82)	13,158 (43.61)
Rural	78,040 (63.89)	21,823 (61.18)	17,016 (56.39)
**Distance to health facility**			
Problem	46,506 (38.07)	11,869 (33.27)	9,632 (31.92)
Not a problem	75,638 (61.93)	23,803 (66.73)	20,542 (68.08)
**Frequency of reading newspaper**			
Not at all	104,773 (85.78)	28,478 (79.83)	24,838 (82.32)
Less than once a week	10,379 (8.50)	4,384 (12.29)	3,210 (10.64)
At least once a week	6,676 (5.47)	2,694 (7.55)	2,022 (6.70)
Almost every day	316 (0.26)	116 (0.33)	104 (0.34)
**Frequency of listening to radio**			
Not at all	58,422 (47.83)	14,709 (41.23)	12,655 (41.94)
Less than once a week	22,999 (18.83)	7,161 (20.07)	6,064 (20.10)
At least once a week	37,975 (31.09)	12,896 (36.15)	10,698 (35.45)
Almost every day	2,748 (2.25)	906 (2.54)	757 (2.510)
**Frequency of watching television**			
Not at all	72,622 (59.46)	19,574 (54.87)	15,317 (50.76)
Less than once a week	14,596 (11.95)	4,537 (12.72)	3,915 (12.97)
At least once a week	31,076 (25.44)	10,288 (28.84)	9,704 (32.16)
Almost every day	3,850 (3.15)	1,273 (3.57)	1,238 (4.10)
**Currently working**			
No	42,688 (34.95)	12,085 (33.88)	10,368 (34.36)
Yes	79,456 (65.05)	23,587 (66.12)	19,806 (65.64)
**Health insurance status**			
No	108,128 (88.53)	31,242 (87.58)	25,868 (85.73)
Yes	14,016 (11.47)	4,430 (12.42)	4,306 (14.27)

Among the women who use modern contraceptives, 31.47% have experienced emotional violence by their husbands/partners, 12.21% have experienced sexual violence, 11.76% have experienced physical violence, and 36.79% have experienced at least one IPV.

Furthermore, among the women who had adequate MHS, 29.49% of the women have experienced emotional violence by their husbands/partners, 9.42% have experienced sexual violence, 10.59% have experienced physical violence, and 34.20% have experienced at least one IPV.

The results further revealed that a higher proportion of the study sample are between 25 and 29 years (20.82%), have no education (33.89%), are in the poorest wealth category (22.30%), live in rural areas (63.89%), have between 3–5 children (39.52%), currently employed (65.05%), reported that distance to health facility is not a problem (61.93%), do not have health insurance (88.53%), do not read newspapers (85.78%), do not listen to the radio (47.83%), and do not watch television (59.46%).

Furthermore, the results from the multicollinearity test showed no signs of multicollinearity as the VIFs were below 5 and the TIs were above 0.20 for all the independent variables [30]. The results are provided in the Appendix (S1 Table).

### Association between IPV and the use of modern contraceptives among women in 25 SSA countries

Table 3 presents results from the multivariable logistic regression analysis on the association between IPV and the use of modern contraceptives among women in 25 SSA countries. Four models were estimated, examining emotional, sexual, and physical violence separately (Models 1–3), and a combined measure of experiencing at least one form of IPV (Model 4).

**Table 3:** Multivariable logistic regression analysis on the association between IPV and the use of modern contraceptives among women in 25 SSA countries.

	Model 1	Model 2	Model 3	Model 4
	Modern Contraceptives	Modern Contraceptives	Modern Contraceptives	Modern Contraceptives
Variables	AOR (95%CI)	AOR (95%CI)	AOR (95%CI)	AOR (95%CI)
**Emotional violence**				
No	Ref			
Yes	1.15 (1.11 - 1.20)***			
**Sexual violence**				
No		Ref		
Yes		1.09 (1.03 - 1.15)**		
**Physical violence**				
No			Ref	
Yes			1.14 (1.07 - 1.20)***	
**At least one IPV**				
No				Ref
Yes				1.15 (1.11 - 1.20)***
**Parity**				
None	Ref	Ref	Ref	Ref
1 – 2 Children	3.68 (3.35 - 4.04)***	3.70 (3.37 - 4.06)***	3.70 (3.36 - 4.06)***	3.68 (3.35 - 4.04)***
3 – 5 Children	5.73 (5.19 - 6.33)***	5.78 (5.23 - 6.38)***	5.77 (5.22 - 6.37)***	5.73 (5.19 - 6.33)***
≥ 6 Children	6.76 (6.07 - 7.54)***	6.83 (6.12 - 7.61)***	6.81 (6.11 - 7.59)***	6.76 (6.06 - 7.53)***
**Age**				
15 - 19	Ref	Ref	Ref	Ref
20 - 24	1.11 (1.02 - 1.20)*	1.11 (1.02 - 1.21)*	1.11 (1.02 - 1.21)*	1.11 (1.02 - 1.20)*
25 - 29	0.96 (0.87 - 1.04)	0.96 (0.88 - 1.05)	0.96 (0.88 - 1.05)	0.96 (0.87 - 1.04)
30 - 34	0.85 (0.77 - 0.93)***	0.85 (0.78 - 0.94)***	0.85 (0.77 - 0.93)***	0.85 (0.77 - 0.93)***
35 - 39	0.70 (0.64 - 0.77)***	0.71 (0.64 - 0.78)***	0.70 (0.64 - 0.78)***	0.70 (0.64 - 0.77)***
40 - 44	0.54 (0.49 - 0.59)***	0.54 (0.49 - 0.60)***	0.54 (0.49 - 0.60)***	0.54 (0.49 - 0.60)***
45 - 49	0.29 (0.26 - 0.33)***	0.29 (0.26 - 0.33)***	0.29 (0.26 - 0.33)***	0.29 (0.26 - 0.33)***
**Educational level**				
None	Ref	Ref	Ref	Ref
Primary	1.55 (1.48 - 1.63)***	1.56 (1.48 - 1.63)***	1.56 (1.48 - 1.63)***	1.55 (1.48 - 1.63)***
Secondary	1.93 (1.82 - 2.05)***	1.93 (1.82 - 2.05)***	1.93 (1.82 - 2.05)***	1.93 (1.82 - 2.05)***
Higher	1.90 (1.71 - 2.12)***	1.89 (1.70 - 2.11)***	1.90 (1.70 - 2.11)***	1.91 (1.71 - 2.12)***
**Wealth**				
Poorest	Ref	Ref	Ref	Ref
Poorer	1.22 (1.16 - 1.29)***	1.22 (1.16 - 1.29)***	1.22 (1.16 - 1.29)***	1.22 (1.16 - 1.29)***
Middle	1.42 (1.34 - 1.50)***	1.42 (1.34 - 1.50)***	1.42 (1.34 - 1.50)***	1.42 (1.34 - 1.50)***
Richer	1.47 (1.38 - 1.57)***	1.47 (1.38 - 1.57)***	1.47 (1.38 - 1.57)***	1.47 (1.38 - 1.57)***
Richest	1.51 (1.39 - 1.63)***	1.50 (1.39 - 1.63)***	1.51 (1.39 - 1.63)***	1.51 (1.39 - 1.64)***
**Residence**				
Urban	Ref	Ref	Ref	Ref
Rural	0.95 (0.90 - 1.00)*	0.95 (0.90 - 1.00)*	0.95 (0.90 - 1.00)*	0.95 (0.90 - 1.00)*
**Distance to health facility**				
Problem	Ref	Ref	Ref	Ref
Not a problem	1.12 (1.07 - 1.16)***	1.12 (1.07 - 1.16)***	1.12 (1.07 - 1.16)***	1.12 (1.08 - 1.16)***
**Frequency of reading newspaper**				
Not at all	Ref	Ref	Ref	Ref
Less than once a week	1.05 (0.99 - 1.12)	1.05 (0.99 - 1.12)	1.05 (0.99 - 1.12)	1.05 (0.99 - 1.12)
At least once a week	1.05 (0.96 - 1.15)	1.05 (0.96 - 1.15)	1.05 (0.96 - 1.15)	1.05 (0.96 - 1.15)
Almost every day	1.66 (1.06 - 2.61)*	1.67 (1.07 - 2.62)*	1.67 (1.07 - 2.63)*	1.66 (1.06 - 2.61)*
**Frequency of listening to radio**				
Not at all	Ref	Ref	Ref	Ref
Less than once a week	1.10 (1.05 - 1.16)***	1.10 (1.05 - 1.16)***	1.10 (1.05 - 1.16)***	1.10 (1.05 - 1.16)***
At least once a week	1.13 (1.09 - 1.18)***	1.13 (1.09 - 1.19)***	1.14 (1.09 - 1.19)***	1.13 (1.08 - 1.18)***
Almost every day	1.28 (1.11 - 1.48)***	1.29 (1.12 - 1.48)***	1.29 (1.12 - 1.49)***	1.28 (1.11 - 1.48)***
**Frequency of watching television**				
Not at all	Ref	Ref	Ref	Ref
Less than once a week	1.14 (1.08 - 1.21)***	1.15 (1.08 - 1.22)***	1.14 (1.08 - 1.21)***	1.15 (1.08 - 1.21)***
At least once a week	1.18 (1.11 - 1.24)***	1.18 (1.11 - 1.24)***	1.18 (1.11 - 1.24)***	1.18 (1.11 - 1.24)***
Almost every day	1.63 (1.41 - 1.89)***	1.64 (1.41 - 1.90)***	1.64 (1.41 - 1.90)***	1.64 (1.41 - 1.90)***
**Currently working**				
No	Ref	Ref	Ref	Ref
Yes	1.25 (1.20 - 1.30)***	1.25 (1.20 - 1.30)***	1.25 (1.20 - 1.30)***	1.25 (1.20 - 1.30)***
**Health insurance status**				
No	Ref	Ref	Ref	Ref
Yes	1.08 (1.00 - 1.17)*	1.08 (1.00 - 1.17)*	1.08 (1.00 - 1.17)*	1.09 (1.00 - 1.17)*
**Constant**	0.01 (0.01 - 0.01)***	0.01 (0.01 - 0.01)***	0.01 (0.01 - 0.01)***	0.01 (0.01 - 0.01)***

**Note:** *N*** = **122,144. AOR = Adjusted Odds Ratio; 95% confidence interval (CI) in parentheses; Ref = Reference group.

*** p < 0.001, ** p < 0.01, * p < 0.05

The results indicate that experience of IPV is associated with a higher likelihood of using modern contraceptives. This is observed for emotional violence (AOR = 1.15, 95% CI: 1.11 - 1.20), sexual violence (AOR = 1.09, 95% CI: 1.03 - 1.15), and physical violence (AOR = 1.14, 95% CI: 1.07 - 1.20). The aggregated results show that women who experienced at least one form of IPV had 15% higher odds of using modern contraceptives (AOR = 1.15, 95% CI: 1.11 - 1.20). Parity, education, wealth, residence, distance to health facility, employment, and health insurance emerged as significant predictors of modern contraceptive use across all four models. However, we report the results from model 4 as that provides an aggregated finding from all the IPV indicators. Regarding parity, women with six or more children had higher odds of contraceptive use compared to those with 1–2 children (AOR = 6.76, 95% CI: 6.06–7.53). Women with secondary education had higher odds compared to those with no education (AOR = 1.93, 95% CI: 1.82–2.05), and women in the richest wealth category had higher odds compared to those in the poorest category (AOR = 1.57, 95% CI: 1.39–1.64). Additionally, women who reported that distance to a healthcare facility is not a problem are more likely to use modern contraceptives (AOR = 1.12, 95% CI = 1.08 - 1.16). Also, women who are currently employed are more likely to use modern contraceptives (AOR = 1.25, 95% CI = 1.20 - 1.30) compared to the unemployed. Moreover, those who have health insurance have higher odds of using modern contraceptives (AOR = 1.09, 95% CI = 1.00 - 1.17) compared to the uninsured. Compared to urban women, rural women are less likely to use modern contraceptives (AOR = 0.95, 95% CI = 0.90 - 1.00).

### Association between IPV and having adequate MHS among women in 25 SSA countries

Table 4 presents results from the multivariable logistic regression analysis on the association between IPV and having adequate MHS among women in 25 SSA countries. Models 1–3 examined emotional, sexual, and physical IPV separately, while Model 4 considered any IPV exposure.

**Table 4:** Multivariable logistic regression analysis on the association between IPV and having adequate MHS among women in 25 SSA countries.

	Model 1	Model 2	Model 3	Model 4
	Adequate MHS	Adequate MHS	Adequate MHS	Adequate MHS
Variables	AOR (95%CI)	AOR (95%CI)	AOR (95%CI)	AOR (95%CI)
**Emotional violence**				
No	Ref			
Yes	0.95 (0.91 - 0.99)*			
**Sexual violence**				
No		Ref		
Yes		0.88 (0.83 - 0.95)***		
**Physical violence**				
No			Ref	
Yes			0.94 (0.88 - 1.00)	
**At least one violence**				
No				Ref
Yes				0.94 (0.90 - 0.98)**
**Parity**				
1 – 2 Children	Ref	Ref	Ref	Ref
3 – 5 Children	0.77 (0.73 - 0.82)***	0.77 (0.73 - 0.82)***	0.77 (0.73 - 0.82)***	0.78 (0.73 - 0.82)***
≥ 6 Children	0.58 (0.54 - 0.63)***	0.58 (0.54 - 0.63)***	0.58 (0.54 - 0.63)***	0.58 (0.54 - 0.63)***
**Age**				
15 - 19	Ref	Ref	Ref	Ref
20 - 24	1.02 (0.93 - 1.11)	1.01 (0.92 - 1.11)	1.02 (0.93 - 1.11)	1.02 (0.93 - 1.12)
25 - 29	1.20 (1.09 - 1.32)***	1.19 (1.08 - 1.32)***	1.20 (1.09 - 1.32)***	1.20 (1.09 - 1.32)***
30 - 34	1.44 (1.29 - 1.60)***	1.43 (1.29 - 1.60)***	1.44 (1.29 - 1.60)***	1.44 (1.29 - 1.60)***
35 - 39	1.60 (1.43 - 1.79)***	1.60 (1.42 - 1.79)***	1.60 (1.43 - 1.79)***	1.60 (1.43 - 1.79)***
40 - 44	1.55 (1.36 - 1.77)***	1.54 (1.35 - 1.76)***	1.55 (1.36 - 1.77)***	1.55 (1.36 - 1.77)***
45 - 49	1.51 (1.28 - 1.78)***	1.50 (1.27 - 1.78)***	1.51 (1.28 - 1.79)***	1.51 (1.28 - 1.78)***
**Educational level**				
None	Ref	Ref	Ref	Ref
Primary	1.43 (1.35 - 1.52)***	1.44 (1.36 - 1.52)***	1.43 (1.35 - 1.52)***	1.44 (1.36 - 1.52)***
Secondary	1.84 (1.72 - 1.97)***	1.84 (1.72 - 1.97)***	1.84 (1.72 - 1.96)***	1.84 (1.72 - 1.97)***
Higher	3.02 (2.62 - 3.49)***	3.02 (2.62 - 3.48)***	3.03 (2.62 - 3.49)***	3.02 (2.62 - 3.48)***
**Wealth**				
Poorest	Ref	Ref	Ref	Ref
Poorer	1.21* (1.13 - 1.28)**	1.21 (1.13 - 1.28)***	1.21 (1.13 - 1.28)***	1.21 (1.13 - 1.28)***
Middle	1.34 (1.26 - 1.44)***	1.34 (1.25 - 1.44)***	1.34 (1.25 - 1.44)***	1.34 (1.26 - 1.44)***
Richer	1.58 (1.46 - 1.71)***	1.58 (1.46 - 1.71)***	1.58 (1.46 - 1.71)***	1.58 (1.46 - 1.71)***
Richest	1.99 (1.80 - 2.20)***	1.99 (1.80 - 2.19)***	1.99 (1.80 - 2.20)***	1.99 (1.80 - 2.19)***
**Residence**				
Urban	Ref	Ref	Ref	Ref
Rural	0.87 (0.82 - 0.94)***	0.87 (0.82 - 0.94)***	0.87 (0.82 - 0.94)***	0.87 (0.82 - 0.94)***
**Distance to health facility**				
Problem	Ref	Ref	Ref	Ref
Not a problem	1.19 (1.13 - 1.24)***	1.19 (1.13 - 1.24)***	1.19 (1.13 - 1.25)***	1.19 (1.13 - 1.24)***
**Frequency of reading newspaper**				
Not at all	Ref	Ref	Ref	Ref
Less than once a week	1.16 (1.07 - 1.26)***	1.16 (1.07 - 1.26)***	1.16 (1.06 - 1.26)***	1.16 (1.07 - 1.26)***
At least once a week	1.04 (0.93 - 1.16)	1.04 (0.93 - 1.16)	1.04 (0.93 - 1.16)	1.04 (0.93 - 1.16)
Almost every day	1.71 (1.08 - 2.71)*	1.70 (1.08 - 2.70)*	1.70 (1.08 - 2.70)*	1.71 (1.08 - 2.71)*
**Frequency of listening to radio**				
Not at all	Ref	Ref	Ref	Ref
Less than once a week	1.10 (1.04 - 1.17)***	1.10 (1.04 - 1.17)***	1.10 (1.04 - 1.17)***	1.10 (1.04 - 1.17)***
At least once a week	1.19 (1.12 - 1.25)***	1.19 (1.12 - 1.25)***	1.18 (1.12 - 1.25)***	1.19 (1.13 - 1.25)***
Almost every day	1.26 (1.08 - 1.48)**	1.26 (1.08 - 1.48)**	1.26 (1.07 - 1.48)**	1.26 (1.08 - 1.48)**
**Frequency of watching television**				
Not at all	Ref	Ref	Ref	Ref
Less than once a week	1.14 (1.06 - 1.22)***	1.14 (1.06 - 1.22)***	1.14 (1.06 - 1.22)***	1.14 (1.06 - 1.22)***
At least once a week	1.32 (1.24 - 1.42)***	1.32 (1.24 - 1.42)***	1.32 (1.24 - 1.42)***	1.32 (1.24 - 1.42)***
Almost every day	1.48 (1.25 - 1.74)***	1.47 (1.25 - 1.73)***	1.47 (1.25 - 1.74)***	1.47 (1.25 - 1.74)***
**Currently working**				
No	Ref	Ref	Ref	Ref
Yes	1.21 (1.15 - 1.26)***	1.20 (1.15 - 1.26)***	1.20 (1.15 - 1.26)***	1.21 (1.15 - 1.27)***
**Health insurance status**				
No	Ref	Ref	Ref	Ref
Yes	1.17 (1.06 - 1.29)**	1.17 (1.06 - 1.29)**	1.17 (1.06 - 1.29)**	1.17 (1.06 - 1.29)**
**Constant**	0.09 (0.08 - 0.11)***	0.09 (0.08 - 0.11)***	0.09 (0.08 - 0.10)***	0.09 (0.08 - 0.11)***

**Note:** *N*** = **76,986. AOR = Adjusted Odds Ratio; 95% confidence interval (CI) in parentheses; Ref = Reference group.

*** p < 0.001, ** p < 0.01, * p < 0.05

The results show that women exposed to emotional (AOR = 0.95, 95% CI: 0.91 - 0.99) or sexual violence (AOR = 0.88, 95% CI: 0.83 - 0.95) are less likely to have adequate MHS. Likewise, women who have experienced physical violence are less likely to have adequate MHS (AOR = 0.94, 95% CI: 0.88–1.00); however, no significant association was observed. In the aggregated model (Model 4), experiencing at least one form of IPV was associated with reduced odds of adequate MHS (AOR = 0.94, 95% CI: 0.90 - 0.98). Other predictors of adequate MHS include parity, age, education, wealth, residence, distance to health facility, employment, and health insurance. The results from Model 4 (the aggregated model) showed that women aged 35 – 39 years had higher odds of adequate MHS compared to those aged 15 – 19 years (AOR = 1.60, 95% CI: 1.43 - 1.79). Women with higher education had greater odds compared to those with no education (AOR = 3.02, 95% CI: 2.62 - 3.48), and women in the richest wealth category had higher odds compared to the poorest (AOR = 1.99, 95% CI: 1.80 - 2.19). Furthermore, women who reported no distance barrier (AOR = 1.19, 95% CI: 1.13 - 1.24), those employed (AOR = 1.21, 95% CI: 1.15 - 1.27), and those with health insurance (AOR = 1.17, 95% CI: 1.06 - 1.29) are more likely to have adequate MHS. Women with six or more children are less likely to have adequate MHS compared to those with 1–2 children (AOR = 0.58, 95% CI: 0.54 - 0.63). Moreover, women in rural areas are less likely to have adequate MHS (AOR = 0.87, 95% CI: 0.82 - 0.94) compared to urban women.

## Discussion

This study investigated the association between various forms of intimate partners violence, such as sexual, physical and emotional violence, and contraceptive usage and MHS among women in 25 sub-Saharan African regions. We noted from our study that 29.2% of women used modern contraceptives, 39.2% received adequate MHS, and 34.3% of women had experienced at least one form of IPV. This new knowledge is important to help guide global efforts against gender-based violence and to further underscore how this influences health-seeking behaviour among victims.

Our study found a significant relationship between IPV and contraceptive use. This finding corroborates previous studies [11,31,32] showing that IVP-exposed women are more likely to adopt modern contraceptive methods. However, findings from a systematic literature review [33] reveals that women who had experienced IPV had about 53% lower odds of using contraception compared to those who did not experience IPV. This equivocal findings from the literature may be attributed to differences in regional focus, measurement approaches, or temporal and cultural contexts across the reviewed studies.

Our findings suggest that some women may resort to covert or self-initiated contraceptive use as a means of preventing further entrapment in violent relationships, particularly in settings where reproductive coercion is common.

Interestingly, we found that, despite increased contraceptive use among IPV-exposed women, these same women were significantly less likely to utilize adequate MHS. This paradox highlights a critical disconnect as women may take steps to avoid unintended pregnancies but still face structural and interpersonal barriers to care during pregnancy and childbirth. Previous studies have similarly documented this duality, noting that while IPV can drive covert contraceptive use as a form of self-protection [10,11], it often constrains access to antenatal, intrapartum, and postpartum care due to fear and lack of partner support [2,17]. These discrepancies highlight the need to view IPV not only as a public health issue but as a structural determinant that shapes women’s health-seeking behaviours across the reproductive lifecycle.

We also observed that education, wealth, health insurance, and rural–urban residence were consistently associated with higher contraceptive and MHS use, even after adjusting for IPV. Women with higher education and income were more likely to overcome barriers to care, even under IPV exposure, suggesting that socioeconomic empowerment may buffer its adverse effects. This finding is supported by previous studies [19,34], which demonstrates that higher educational attainment enhances health literacy, decision-making autonomy, and access to information, all of which contribute to improved reproductive health outcomes even under challenging circumstances. Wealth and insurance coverage reduce financial barriers, enabling women to access services independently of their partners’ control, which is critical in contexts where IPV survivors may face economic disempowerment. This association has been consistently documented in multi-country analyses across SSA, where higher household wealth is positively linked with increased uptake of antenatal care, facility-based delivery, and postnatal services because women from richer households are better positioned to afford both direct and indirect costs of care [35–38].

Similarly, in sub-Saharan Africa, where health systems are often under-resourced, health insurance plays a critical role in reducing financial barriers to antenatal care, facility-based delivery, and postnatal services. Evidence shows that coverage increases service utilization by alleviating out-of-pocket costs and promoting continuity of care [36,39]. For IPV survivors, insurance can be particularly empowering, enabling independent healthcare access without relying on partners’ financial support. This finding is particularly important in the context of sub-Saharan Africa, as it highlights the need to prioritise financial barrier–free care for women in the broader pursuit of achieving the Sustainable Development Goals (SDGs), particularly those related to health, gender equality, and poverty reduction.

Regular exposure to media such as radio, television and newspapers was associated with increased uptake of modern contraceptives and MHS in SSA. This finding supports previous studies that media exposure enhances awareness of reproductive health services, challenging harmful norms, and empowering women to seek care, particularly in resource-limited and rural settings [19,40]. This association calls for stronger alignment between national health communication strategies and media infrastructure emphasizing local languages, culturally sensitive content, and programming tailored to underserved groups. Strengthening health messaging through public and community media can bridge the awareness gap, improve service uptake and contribute to broader goals under the Sustainable Development Goals (SDGs), particularly SDG 3 (good health and well-being) and SDG 5 (gender equality).

### Strengths and limitations

A key strength of this study lies in its use of large, nationally representative and up-to-date datasets drawn from 25 sub-Saharan African countries, enhancing the generalizability of the findings across the region. The use of data from the Demographic and Health Surveys (DHS) provides the analysis with standardized measures of both intimate partner violence (IPV) and reproductive health service utilization, enabling robust comparisons across countries. Furthermore, the inclusion of multiple forms of IPV, including emotional, physical, and sexual violence, provides a more nuanced understanding of how violence affects women’s reproductive health behaviours across different contexts.

Another strength is the study’s analytical approach, which controlled for a wide range of individual, household, and community-level covariates. This allowed for a clearer assessment of the associations between IPV and reproductive health outcomes and provided insight into how structural factors such as education, wealth, and health insurance influence these relationships.

However, the study has several limitations. Firstly, its cross-sectional design limits the ability to infer causality, making it difficult to establish the temporal relationship between IPV exposure and health service utilization. Secondly, IPV is a highly sensitive issue, and the reliance on self-reported data may have resulted in underreporting due to stigma, fear, or social desirability bias. Furthermore, while the study examined several structural variables, other important factors, such as quality of care, provider attitudes, or community-level gender norms, could not be included due to data constraints. Lastly, differences in data collection periods across countries may introduce inconsistencies in the pooled analysis.

## Conclusion

This study highlights the complex relationship between intimate partner violence and women’s reproductive health behaviours in sub-Saharan Africa. Our cross-country, multi-dimensional analysis reveals that while IPV exposure is associated with increased use of modern contraceptives, it is also linked to reduced uptake of adequate MHS, pointing to a critical gap in the reproductive health continuum.

The findings advance existing literature by offering large-scale, cross-national evidence that IPV remain both a barrier and, paradoxically, a driver of certain reproductive choices.

Policy responses must integrate IPV prevention into reproductive health strategies, strengthen health communication through media, and ensure women’s access to comprehensive and affordable MHS. Doing so is essential for achieving Sustainable Development Goals 3 (health) and 5 (gender equality) and for safeguarding the health and autonomy of women experiencing violence.

## Supporting information

S1 Table Collinearity diagnostics. (DOCX)
