# Supplementary material for: The shadow of violence: How intimate partner violence shapes contraceptive and maternal health service use across 25 African countries
Source: PLOS Glob Public Health. 2025 Nov 20;5(11):e0005470. doi: 10.1371/journal.pgph.0005470 (PMC12633915; doi:10.1371/journal.pgph.0005470)
Supplement: S1 Table — (DOCX) [file pgph.0005470.s001.docx]

**S1 Table: Collinearity Diagnostics**

| **Variables** | **Variance Inflation Factor** | **Tolerance Indices** |
| --- | --- | --- |
| Emotional violence | 1.24 | 0.81 |
| Sexual violence | 1.17 | 0.86 |
| Physical violence | 1.24 | 0.81 |
| Parity | 1.72 | 0.58 |
| Age | 1.62 | 0.62 |
| Educational level | 1.53 | 0.65 |
| Wealth | 1.82 | 0.55 |
| Residence | 1.58 | 0.63 |
| Distance to health facility | 1.11 | 0.90 |
| Frequency of reading newspaper | 1.24 | 0.81 |
| Frequency of listening to radio | 1.18 | 0.85 |
| Frequency of watching television | 1.63 | 0.61 |
| Currently working | 1.05 | 0.95 |
| Health insurance status | 1.07 | 0.94 |
